# Cosinor modelling of seasonal variation in 25-hydroxyvitamin D concentrations in cardiovascular patients in Norway

**DOI:** 10.1038/ejcn.2015.200

**Published:** 2015-11-25

**Authors:** E Degerud, R Hoff, O Nygård, E Strand, D W Nilsen, J E Nordrehaug, Ø Midttun, P M Ueland, S de Vogel, J Dierkes

**Affiliations:** 1 Department of Clinical Medicine, University of Bergen, Bergen, Norway; 2 The Institute of Basic Medical Sciences, University of Oslo, Oslo, Norway; 3 Department of Clinical Science, University of Bergen, Bergen, Norway; 4 Department of Heart Disease, Haukeland University Hospital, Bergen, Norway; 5 Department of Cardiology, Stavanger University Hospital, Stavanger, Norway; 6 Bevital AS, Bergen, Norway; 7 Laboratory of Clinical Biochemistry, Haukeland University Hospital, Bergen, Norway; 8 Department of Global Public Health and Primary Care, University of Bergen, Bergen, Norway

## Abstract

**Background/Objectives:**

Seasonal variation may reduce the validity of 25-hydroxyvitamin D (25OHD) as a biomarker of vitamin D status. Here we aimed to identify potential determinants of seasonal variation in 25OHD concentrations and to evaluate cosinor modelling as a method to adjust single 25OHD measurements for seasonal variation.

**Subjects/Methods:**

In Caucasian cardiovascular patients (1999–2004), we measured 25OHD by liquid chromatography tandem mass spectrometry in 4116 baseline and 528 follow-up samples. To baseline values, we fitted a cosinor model for monthly concentrations of 25OHD. Using the model, we estimated each patient's adjusted annual 25OHD value. Further, we studied how covariates affected the annual mean 25OHD concentration and seasonal variation of the study cohort. To evaluate the model, we predicted follow-up measurements with and without covariates and compared accuracy with carrying forward baseline values and linear regression adjusting for season, common approaches in research and clinical practice, respectively.

**Results:**

The annual mean (59.6 nmol/l) was associated with participants' age, gender, smoking status, body mass, physical activity level, diabetes diagnosis, vitamin D supplement use and study site (adjusted models, *P*<0.05). Seasonal 25OHD variation was 15.8 nmol/l, and older age (>62 years) was associated with less variation (adjusted model, *P*=0.025). Prediction of follow-up measurements was more accurate with the cosinor model compared with the other approaches (*P*<0.05). Adding covariates to cosinor models did not improve prediction (*P*>0.05).

**Conclusions:**

We find cosinor models suitable and flexible for analysing and adjusting for seasonal variation in 25OHD concentrations, which is influenced by age.

## Introduction

In countries located increasingly distant from the Earth's equator, the population concentration of 25-hydroxyvitamin D (25OHD) tends to follow changes in the ultraviolet B radiation from the sun.^1, 2, 3, 4, 5, 6^ The use of a single measurement of 25OHD to assess vitamin D status and categorise individuals according to vitamin D status may introduce a systematic bias and should be accounted for when analysing the relationship between 25OHD concentrations and a specific outcome. An alternative is to adjust the relationship between 25OHD concentrations and the outcome for season by including season as a covariate in inferential statistical analysis. Another to adjust the measured 25OHD values for seasonality before descriptive analyses, resulting in a season-specific quartile^7, 8^ or a unique value for each person. Unique values can be obtained with different approaches, including simple computation^9^ or modelling.^10, 11^ An example of a computation is adding to each participant's measured value, the mean difference between the study sample and the participants with blood measurements taken in the same month as that of the individual.^9^ An example of a modelling approach is the use of a cosinor model, which assumes that the seasonal variation follows a sinusoidal pattern.^11^ However, the degree of seasonal variation may be influenced by other factors, such as pigmentation of the skin, age, gender and body mass. Among the methods mentioned above, the cosinor model is the only one that allows identification of and adjustment for potential determinants of seasonal variation in 25OHD concentration. Cosinor models may increase the accuracy of the assessment of vitamin D status in observational studies. It may also help clinicians identify patients at risk of developing vitamin D insufficiency across seasons.

The purpose of this study was to use cosinor models to identify potential determinants of seasonal variation in 25OHD concentrations and to evaluate the model as an approach to adjust single measurements of 25OHD for seasonal variation. Previous evaluations of the model as an approach to adjust for seasonal variation were conducted in New Zealand^10^ and North America,^11^ and we aimed to contribute with data from Europe. We used baseline measurements of 25OHD from 4116 cardiovascular patients and follow-up measurements from 271 nested patients.

## Subjects and methods

The Bergen Coronary Angiography Cohort (BECAC) included patients who underwent elective coronary angiography at Haukeland University Hospital, Bergen, Norway (60° North), between January 1999 and April 2004. Patients who agreed to participate in BECAC were also asked to participate in Western Norway B-Vitamin Intervention Trial (WENBIT), a two-centre randomised controlled trial.^12^ The second WENBIT centre was Stavanger University Hospital, Stavanger, Norway (59° North). From the overlapping samples in WENBIT and BECAC, we included patients (*n*=4116) with suspected or verified stable angina pectoris and available 25OHD concentrations from baseline plasma samples. A flow chart is provided (Supplementary Figure 1).

One or two follow-up measurements of 25OHD, in total 528, were available from 271 WENBIT patients who also participated in a nested substudy.^13^ The substudy aimed to study progression of coronary artery disease and only recruited WENBIT participants enrolled at Haukeland University Hospital with coronary artery disease verified by the first angiography. Follow-up measurements were collected at two substudy exams ~1 month and 1 year after baseline. The studies were conducted according to the principles of the Declaration of Helsinki and approved by the Regional Committee for Medical and Health Research Ethics and the Norwegian Data Inspectorate. All participants gave broad written consent for the use of the collected data in future research.

### Blood sampling and measurement of 25OHD

Blood samples were collected between 1999 and 2005 by study personnel and stored at −80 °C. Plasma concentrations of 25OHD2 and 25OHD3 were analysed between 2011 and 2012 using liquid chromatography tandem mass spectrometry at Bevital AS (www.bevital.no), which is certified by the Vitamin D External Quality Assessment Scheme (www.deqas.org).^14^ 25OHD is stable during long-term storage in plasma samples and after repeated freeze and thawing cycles.^15^ Measurements below the lower limit of quantification (6.6 nmol/l) were excluded for both 25OHD3 (*n*=1) and 25OHD2 (*n*=4037 and *n*=268 at both follow-up exams). One participant with very high 25OHD2 was excluded. The concentrations of 25OHD2 and 25OHD3 were summed to reflect total 25OHD concentration.

### Covariates

Anthropometry and blood pressure measurements were performed by study personnel. Smoking was defined as a self-reported smoker or stopped smoking ⩽90 days ago or a plasma cotinine concentration >85 nmol/l (~15 ng/ml).^16^ Plasma cotinine was measured by liquid chromatography tandem mass spectrometry at Bevital AS.^17^ Data on supplement consumption and physical activity level were based on self-report. Diabetes mellitus was defined as a pre-existing diagnosis of either type I or II. Hypertension was defined by systolic blood pressure >140 mm Hg and/or diastolic blood pressure >90 mm Hg and/or the use of antihypertensive drug. Hypercholesterolaemia was defined as familial hypercholesterolaemia or untreated total serum cholesterol ⩾6.5 mmol/l=251.4 mg/dl. The estimated glomerular filtration rate was calculated using the formula suggested by the Chronic Kidney Disease Epidemiology Collaboration.^18^ Serum concentrations of apolipoprotein A-1 and apolipoprotein B were analysed with the Hitachi 917 system (Roche Diagnostics GmbH, Mannheim, Germany). The measurement of serum C-reactive protein was performed with a latex, high-sensitivity assay (Behring Diagnostics, Marburg, Germany). The angiographic extent of coronary artery disease was assessed by trained cardiologists. This variable is described as the aggregated number of stenotic blood vessels (⩾50% luminal narrowing). To reflect months during which sun exposure results in significant or negligible synthesis of vitamin D, we defined the four seasons of blood draw as January–March, April–June, July–September and October–December. The dark (October–March) and bright (April–September) periods were defined based on the same reasoning.

### Statistical analysis

To analyse the seasonal variation of 25OHD concentrations, we fitted cosinor models to baseline measurements, choosing month of measurement to represent time. The cosinor model consists of fitting a linear regression where the 25OHD measurements are regressed onto a sine and a cosine term of transformations of the time variable.^19^ Taken together, the terms give a linear representation of a sine curve with amplitude and a phase, which can be used to describe a seasonal pattern. From the regression output, the coefficient for the intercept is the mean level of the sine curve and thus an estimate of the annual mean 25OHD concentration of the study sample. Confidence intervals for the intercept were calculated directly from the regression output. The amplitude, which is defined as the distance from the mean to the highest (peak) or lowest (trough) location of the curve, provides an estimate of seasonal variation. The phase is the location of the peak on the x axis, equivalent to the month where the 25OHD concentration is highest. Because of symmetry the trough will be 6 months apart from the peak. Both amplitude and phase can be calculated from the regression coefficients for the sine and cosine term.^19^ Standard errors and corresponding confidence intervals are calculated with the Delta method. The distance from the highest to the lowest point of the sine curve (peak-trough), equivalent to twice the amplitude, is an estimate of average total seasonal variation.

To investigate associations of the annual mean 25OHD concentration with patient characteristics, we included covariates in the model. Including a covariate does not alter the shape of the curve but rather shifts it up or down. We used simple and multivariate Wald tests directly on regression coefficients of interest to test whether any differences in annual mean were due to chance. To investigate whether seasonal variation differs according to patient characteristics, we added interaction terms between covariates and the sine and cosine terms, as both the amplitude and the phase of the sine curve can change according to covariate values. Simple and multivariate Wald tests were performed using estimated amplitudes and standard errors calculated with the Delta method. Covariate association with both annual mean and amplitude of 25OHD was investigated for gender, age, study site, smoking, body mass index, physical activity, estimated glomerular filtration rate, C-reactive protein, vitamin D supplement use and a diagnosis of diabetes mellitus. We first investigated each covariate in an unadjusted model without other covariates and then in a model adjusted for age, gender, study site, body mass index and smoking. Covariates in adjusted models were selected because of their relationship with the mean annual 25OHD or the seasonal variation of 25OHD in unadjusted models, provided covariate data were available for most participants.

We evaluated the cosinor model fitted to the 4116 baseline measurements by assessing how accurately the model predicted follow-up measurements from a nested subgroup of 271 participants who attended either one or two follow-up exams. For each patient, we predicted the 25OHD concentration at the months of follow-up by calculating patient-specific sine curves from the cosinor model. The height of the curve was set to match the patient's observed value at baseline. The shape of the curve was determined by the patient's covariate values. Predicted values for different months could then be derived from the curve. The predictive performance was assessed by calculating squared errors, where the errors are defined as the difference between predicted values and measured values at follow-up. We compared the predictions of a cosinor model with carrying baseline values forward and predictions of a linear regression adjusting for the season of blood draw. We also investigated whether adding covariates to the cosinor model improved prediction. Comparisons were carried out with paired *t*-tests applied to the mean squared errors.

To adjust measured 25OHD values for seasonal variation, we added individual deviations from the fitted sine curve to the annual mean of the study sample, thereby calculating an annual value for each participant. If we impose a threshold in 25OHD concentration for what is considered sufficient or insufficient, participants may be classified differently depending on whether the annual or the measured value is used. Participants were categorised as either vitamin D sufficient (⩾50 nmol/l) or insufficient (<50 nmol/l) using both measured values and annual values estimated from cosinor models with and without covariates and linear regression adjusting for season of blood draw. We then calculated the number and proportion of participants who were reclassified. The agreement was assessed considering the year as a whole and within the dark and bright period separately.

*P*-values are two-sided and the level of significance set at a value of *P*<0.05. We did not adjust for multiple comparisons. All statistical analyses were performed in R (version 3.1.1)^20^ using the packages season^21^ (version 0.3–4) and cosinor (version 1.1).

## Results

Table 1 shows descriptive statistics at baseline of all participants (*n*=4116) and a subgroup (*n*=271) with available follow-up measurements of 25OHD from participation in a nested substudy. Because of substudy inclusion criteria nested participants differed from the study cohort for the extent of coronary artery disease, statin treatment coverage and study site, which in turn may have resulted in a difference in measured 25OHD concentration (66 versus 59 nmol/l, *P*<0.001).

**Figure 1:**
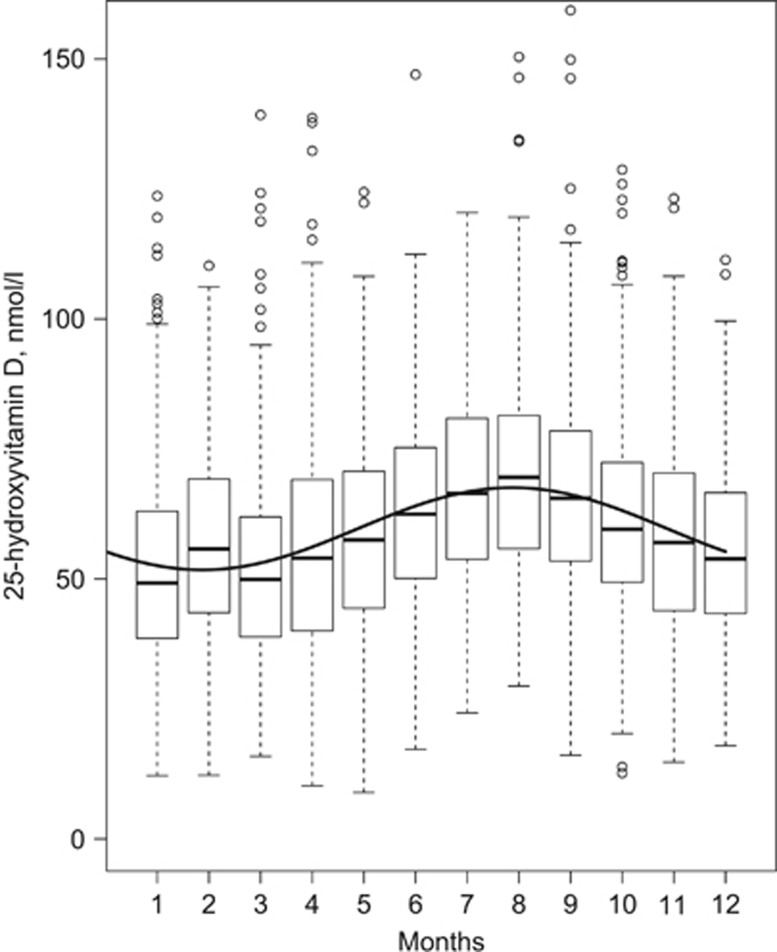
Seasonality of 25OHD concentrations in 4116 participants at baseline. Boxplots show median 25OHD concentration as dark horizontal lines, hinges encompass the 25th and 75th percentiles, whiskers either show maximum value or 1.5 times the interquartile range, and values beyond are plotted as points. The y axis is truncated at 155 nmol/l. The overlaid curve is from an unadjusted cosinor model and shows the annual mean 25OHD concentration according to month of the year.

Figure 1 shows the measured 25OHD concentrations at baseline according to the month of blood sampling. The months with lowest and highest measured mean concentration were March (51.7±18.5 nmol/l) and August (70.4±20.3 nmol/l), respectively. A fitted line from an unadjusted cosinor model is overlaid on the measured values. The annual mean 25OHD concentration derived from the model was 59.6 nmol/l, with a trough occurring between January and February and a peak between July and August. Total difference in annual mean 25OHD concentration from peak to trough was 15.8 nmol/l (amplitude: 7.9 nmol/l), equivalent to 26.5% of annual mean. Assuming that the estimated seasonal variation is true for all participants, a concentration of 65.8 nmol/l would thus be required in August to remain vitamin D sufficient (>50 nmol/l) in February.

The associations between covariates and mean annual concentration of 25OHD and seasonal variation are presented in Table 2. In both unadjusted and adjusted analyses, older age (>62 years), higher physical activity levels and regular consumption of vitamin D supplements were associated with a higher annual mean 25OHD concentration. A lower annual mean was associated with female gender, study site, smoking, adiposity and having a diagnosis of diabetes mellitus. The mean difference (95% confidence interval) in annual mean 25OHD between obese and normal weight participants was −6.4 (−8.4, −4.5) nmol/l in a sensitivity analysis (*n*=3027), which further adjusted for vitamin D supplement consumption and physical activity.

In unadjusted analyses, smoking was associated with more seasonal variation in 25OHD concentration, whereas female gender, older age and a regular consumption of vitamin D supplements were associated with less seasonal variation (Table 2). In adjusted analyses, only the association with age remained statistically significant. The mean peak-trough 25OHD concentration was 19 nmol/l for participants below 62 years of age and 12.3 nmol/l for participants above 62 years of age. To remain vitamin D sufficient (>50 nmol/l) at the time of the seasonal trough, a concentration of 69 and 62.3 nmol/l would thus be required during the peak for participants below and above 62 years of age, respectively.

Time from baseline to the first and second follow-up measurement varied between participants (Supplementary Table 1). Predictions of 25OHD concentrations at the time of follow-up measurements from an unadjusted cosinor model were more accurate than carrying forward baseline values and compared with predictions from a linear regression model adjusted for season of blood draw (*P*<0.05; Table 3). The proportion of predictions that differed <10 nmol/l from measured values was 59% with an unadjusted cosinor model, 55% when carrying forward baseline values and 54% with the linear model adjusting for season. Because of the association of age with seasonal variation in 25OHD concentrations in the adjusted model, we assessed whether adding age to a cosinor model would change the accuracy of predictions. We also investigated the accuracy of a multivariate model with covariates associated with seasonal variation in univariate models (age, gender, smoking and vitamin D supplement consumption). No difference in predictive accuracy was observed between the simple cosinor model and cosinor models with covariates.

Table 4 shows participants categorised as vitamin D sufficient or insufficient according to measured 25OHD and annual 25OHD values from an unadjusted cosinor model and reclassification across this threshold of 50 nmol/l. Overall, 10.4% of participants were reclassified across the threshold. Reclassification of a cosinor model adjusted for age (10.6%), multivariate cosinor model (10.8%) and linear regression with dummy variables for season (7.8%) are presented separately (Supplementary Table 2).

## Discussion

Our study found several patient characteristics that associated with the mean annual 25OHD concentration of the study sample. The characteristic that most strongly associated with a higher concentration was regular consumption of vitamin D supplements, which was predominantly (94%) in the form of cod liver oil (~10 μg vitamin D3 per teaspoon, 5 ml). The characteristic that most strongly associated with a lower concentration was adiposity, which is an established relationship^22^ that may reflect retention of vitamin D in adipose tissue,^23^ extracellular pool size,^24^ lifestyle factors or a combination. The amount of vitamin D required to reach a target concentration of 25OHD in clinical trials is proportional to the increase in body weight.^25^ Hence, a person of 100 kg requires twice as much vitamin D as a person of 50 kg to sustain the same 25OHD concentration. A more comprehensive adjustment for lifestyle did only slightly attenuate this association. Seasonal 25OHD variation was lower in participants of older age (⩾62 years), potentially reflecting age-dependent reductions in the capacity to synthesis vitamin D from ultraviolet B exposure.^26, 27^ Some^10, 11^ but not all^4^ studies using cosinor modelling to assess this association reported similar findings, and a reverse association was observed in younger populations.^6^ We hypothesise that retirement provides opportunity for sun exposure during a longer period of the year, for example, by an increase in recreational physical activity,^28^ and thereby reduces the compulsion for exaggerated and compensatory sun exposure during the summer. Such behaviours may also explain why we observe that older participants have slightly higher and more stable vitamin D status throughout the year than younger participants.

Cosinor modelling has to our knowledge been evaluated previously in two multiethnic cohorts of patients in New Zealand^10^ (37°S) and community-living subjects in North America^11^ (~34 to ~45°N), as a method to adjust 25OHD concentration for seasonal variation and predict future concentrations. The current study provides data from a North European (59–60°N) cohort of Caucasian patients. We observed that predictions from an unadjusted cosinor model corresponded more accurately to follow-up values than if we carried forward baseline values or predicted from linear regression adjusting for season. Similar findings were observed in North America, whereas the study from New Zealand did not compare with other approaches. Using the unadjusted cosinor model, the percentage of predictions within ±10 nmol/l (4 ng/ml) of follow-up values was 59% in our cohort, similar to 57% in North America,^11^ and somewhat less accurate than in New Zealand (74%).^10^ In the North American study, age was associated with seasonal variation, but age-specific sine curves did not result in a more accurate prediction of future vitamin D status nor did any other covariate-specific adjustments. Hence, a crude model without additional covariates seems to be adequate when adjusting for seasonal variation in different populations, continents and latitudes.

Baseline values did more accurately correspond to follow-up values than predictions from a linear model adjusting for season, in contrast to findings in the North American study.^11^ We believe that this difference could reflect the time interval between repeated measurements. In our study, the first follow-up measurement occurred within 1 month following the baseline month for 66% of the 271 participants. In terms of prediction, the close proximity may have favoured carrying forward baseline values, as 25OHD concentrations from over a short time period are likely to correlate. The North American study had repeated measurements more equally spaced throughout the year, which could be to the advantage of the linear regression model. There are other modelling approaches that could be modified for analysis of seasonal variation. However, our aim was not to identify the optimal approach by assessing all possibilities but to evaluate the suitability of the cosinor model in comparison with common approaches in epidemiology and clinical practice.

The proportion of participants classified as vitamin D insufficient was 34.1% according to measured values and 32.5% according to annual values from an unadjusted cosinor model. In comparison with New Zealand^10^ (48% and 63%, respectively), this change was moderate. However, the proportion reclassified across the threshold of sufficiency was 10.4% (*n*=429), which is higher than 7.1% reclassification in North America.^11^ The impact of adjusting for seasonality was more pronounced with cosinor models than with linear regression with dummy variables for season (7.8%). We also consider these adjustments to be more reliable, if we regard performance in predicting future vitamin D status as an indirect measurement of accuracy in the adjusted values.

Cosinor modelling provides a flexible framework for analysing and adjusting for seasonality of 25OHD. It compares favourable in terms of predictive performance to linear regression with dummy variables for season and to simply carry forward baseline values, which are common approaches in epidemiology and clinical practice, respectively. Age was a determinant of seasonal variation in 25OHD but did not enhance prediction of future vitamin D status in comparison with a simple cosinor model.

## Figures and Tables

**Table 1:** Baseline characteristics of the study sample and nested subgroup

*Variable*	*All participants (*n*=4116)*	*Nested participants with follow-up measurements (*n*=271)*
25-Hydroxyvitamin D level (nmol/l)	59.4±20.3	65.9±21.4
Age (years)	61.8±10.4	60.8±10.2
Male sex, *n* (%)	2960 (71.9)	218 (80.4)
		
*Study site,* n *(%)*		
Bergen	3369 (81.9)	271 (100)
Stavanger	747 (18.1)	—
Smoking, *n* (%)	1301 (31.7)	82 (30.3)
Body mass index (kg/m^2^)	26.8±4.0	26.9±3.4
C-reactive protein ⩾10 mg/l, *n* (%)	278 (6.8)	13 (4.8)
Physical activity ⩾2 h per week, *n* (%)	2135 (68.8)	185 (70.3)
Vitamin D supplements regularly or daily, *n* (%)	1191 (33.6)	98 (37.5)
Diabetes mellitus type 1 or 2, *n* (%)	491 (11.9)	29 (10.7)
Hypertension, *n* (%)	1928 (46.8)	121 (44.6)
Systolic blood pressure, mm Hg	141±20.8	142±21.6
Estimated glomerular filtration rate, ml/min per 1.73^2^	87.8±17.2	90.9±14.6
Hypercholesterolaemia, *n* (%)	2228 (57.9)	162 (62.8)
Statin therapy, *n* (%)	2982 (72.4)	226 (83.4)
		
*Extent of coronary artery disease*		
No stenotic vessels	1035 (25.1)	—
One vessel	952 (23.1)	137 (50.6)
⩾Two vessels	2129 (51.7)	134 (49.4)

Continuous variables are presented as mean±s.d. and categorical variables as numbers (*n*) and percentages (%). Missing values: smoking (*n*=7), BMI (*n*=3), systolic blood pressure (*n*=12), hypercholesterolaemia (*n*=267), physical activity level (*n*=1015), C-reactive protein (*n*=1), estimated glomerular filtration rate (*n*=3) and vitamin D supplement use (*n*=523).

Abbreviation: BMI, body mass index.

**Table 2:** Covariate associations with annual mean 25OHD concentration (nmol/l) and its seasonal variation^a^

*Variables*	*Unadjusted*				*Adjusted*^b^			
	*Difference in annual mean 25OHD*	P*-value*^c^	*Difference in seasonal 25OHD variation*^d^	P*-value*	*Difference in annual mean 25OHD*	P*-value*	*Difference in seasonal 25OHD variation*	P*-value*
*Gender (n=4116)*								
Male	Ref.	—	—	—	—	—	—	—
Female	−1.3 (−2.7, 0.0)	0.052	−4.8 (−8.7, −1.0)	0.014	−2.2 (−3.5, -0.9)	0.001	−4.2 (−10.3, 2.0)	0.184
								
*Age (n=4116)*								
<62 years	Ref.	—	—	—	—	—	—	—
⩾62 years	3.3 (2.1, 4.5)	<0.001	−6.7 (−10.2, −3.4)	<0.001	1.9 (0.7, 3.2)	0.002	−6.3 (−11.8, −0.8)	0.025
								
*Study site (n=4116)*								
Bergen	Ref.	—	—	—	—	—	—	—
Stavanger	−5.2 (−6.8, −3.7)	<0.001	2.1 (−2.4, 6.6)	0.357	−5.3 (−6.8, −3.8)	<0.001	1.5 (−5.1, 8.2)	0.651
								
*Smoking (n=4109)*								
No	Ref.	—	—	—	—	—	—	—
Yes	−3.4 (−4.7, −2.2)	<0.001	4.9 (1.2, 8.6)	0.009	−3.4 (−4.7, −2.1)	<0.001	3.2 (−2.5, 8.9)	0.269
								
*BMI (n=4113)*								
<25	Ref.	—	—	—	—	—	—	—
25–30	−2.4 (−3.7, −1.1)	<0.001	0.1 (−3.7, 3.9)	0.958	−2.3 (−3.6, −1.0)	0.001	−1.0 (−6.3, 4.3)	0.703
>30	−8.9 (−10.6, −7.2)	<0.001	−2.0 (−6.9, 2.8)	0.401	−8.6 (−10.3, −6.9)	<0.001	−2.2 (−8.2, 3.9)	0.481
								
*Physical activity level (n=3101)*								
⩽1 h per week	Ref.	—	—	—	—	—	—	—
⩾2 h per week	5.5 (4.0, 7.0)	<0.001	−3.1 (−7.3, 1.1)	0.152	4.1 (2.6, 5.6)	<0.001	−3.4 (−10.7, 3.9)	0.358
								
*eGFR (ml/min per 1.73 m^2^) (n=4113)*								
>60	Ref.							
⩽60	4.2 (1.8, 6.7)	0.001	−6.2 (−13.5, 1.2)	0.099	2.4 (−0.1, 4.8)	0.056	−2.5 (−11.4, 6.4)	0.578
								
*CRP (mg/l) (n=4115)*								
⩽10	Ref.	—	—	—	—	—	—	—
>10	−2.6 (−5.0, −0.2)	0.035	−4.9 (−11.9, 2.1)	0.167	−2.1 (−4.4, −0.3)	0.084	−4.3 (−12.9, 4.3)	0.325
								
*Vitamin D supplements (n=3593)*								
Never or seldom	Ref.	—	—	—	—	—	—	—
Regularly or daily	9.5 (8.2, 10.8)	<0.001	−5.1 (−8.8, −1.4)	0.008	8.4 (7.1, 9.6)	<0.001	−4.2 (−10.7, 2.4)	0.210
								
*Diabetes mellitus (n=4116)*								
No	Ref	—	—	—	—	—	—	—
Yes	−2.8 (−4.7, −1.0)	0.003	−0.5 (−5.9, 4.8)	0.845	−1.9 (−3.7, −0.3)	0.047	−0.2 (−7.6, 7.3)	0.968

Numbers presented are mean differences with 95% CIs for the annual concentrations of 25OHD and its seasonal variation according to covariate levels.

Abbreviations: BMI, body mass index; CI, confidence interval; CRP, C-reactive protein; eGFR, estimated glomerular filtration rate; 25OHD, sum of 25-hydroxyvitamin D2 and D3.

a Among 4116 Caucasian patients with suspected stable angina pectoris.

b Adjusted model included age, gender, study site, BMI and smoking (*n*=4006). Remaining relationships were assessed in separate models, which also contained these adjustments.

c *P*-values from univariate or multivariate Wald tests.

d Seasonal variation defined as the difference from peak (high) to trough (low) 25OHD concentration of sine curve for a given level of a covariate.

**Table 3:** Accuracy of methods in predicting 528 follow-up measurements of 25-hydroxyvitamin D from 271 participants

*Method*^a^	*Correlation (95% CI)*^b^	*MSE*	*RMSE*	*S.d.*	*Method comparison*^c^ P*-value*
*Cosinor*					
Unadjusted	0.77 (0.74, 0.81)	198	14.1	21.4	Ref.
Plus age	0.77 (0.73, 0.80)	199	14.1	21.4	0.427
Multivariate	0.77 (0.73, 0.80)	201	14.2	21.4	0.331
Baseline value carried forward	0.75 (0.71, 0.78)	221	14.8	21.4	0.001
Linear regression adjusted for season	0.71 (0.67, 0.75)	246	15.7	21.2	<0.001

Abbreviations: CI, confidence interval; MSE, mean squared error; RMSE, root mean squared error.

MSE is the difference between predicted and measured values squared; RMSE is the square root of mean squared errors; s.d. of predicted values.

a Missing values (*n*): linear regression (*n*=2), cosinor unadjusted (*n*=2), plus age (*n*=2), multivariate model with age, gender, smoking and vitamin D supplement consumption (*n*=6).

b Pearson's product-moment correlation coefficient with 95% CI of predicted or baseline values with measured values at follow-up.

c Differences in predictive accuracy of methods were tested with paired *t*-tests on squared errors.

**Table 4:** Classification of subjects according to vitamin D status^a^

*Measured 25OHD concentration*	*Annual 25OHD concentration*		*Reclassified*
	*⩾50 nmol/l*	*<50 nmol/l*	
All year (*n*=4116)	n	n	%
⩾50 nmol/l	2531	183	6.7
<50 nmol/l	246	1156	17.5
Total			10.4
			
*Dark period (n=2214)*			
⩾50 nmol/l	1271	38	2.9
<50 nmol/l	214	691	23.6
Total			11.4
			
*Bright period (n=1902)*			
⩾50 nmol/l	1260	145	10.3
<50 nmol/l	32	465	6.4
Total			9.3

Abbreviation: 25OHD, sum of 25-hydroxyvitamin D2 and D3.

Dark and bright periods were defined as months of negligible or significant vitamin D synthesis from sun exposure, respectively. Dark period ranges from October to March and bright period from April to September.

a Obtained from measured concentrations of 25OHD (nmol/l) and annual concentrations derived from an unadjusted cosinor model.
