# Supplementary material for: Cosinor modelling of seasonal variation in 25-hydroxyvitamin D concentrations in cardiovascular patients in Norway
Source: Eur J Clin Nutr. 2015 Nov 25;70(4):517–22. doi: 10.1038/ejcn.2015.200 (PMC4827012; doi:10.1038/ejcn.2015.200)
Supplement: Supplementary Information [file ejcn2015200x1.pdf]

Online Supplemental Material
Supplemental Figure 1.
Flow of participants with verified or suspected stable angina pectoris who underwent coronary angiography at two hospitals located in Bergen and Stavanger between 2001 and 2004.

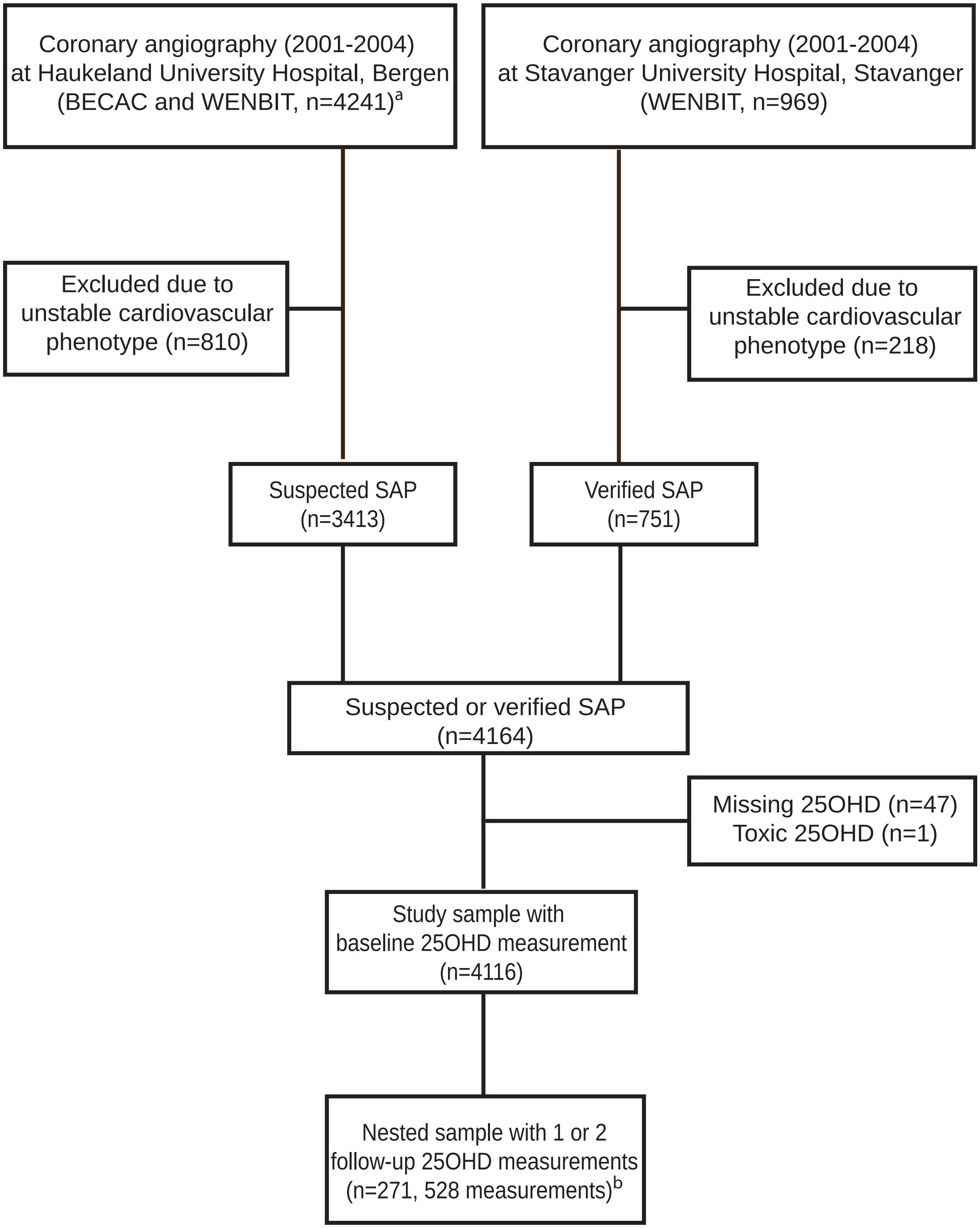


[a] BECAC = Bergen Coronary Angiography Cohort
WENBIT = Western Norway B-Vitamin Intervention Trial
The BECAC was used as the source population for recruitment to WENBIT in Bergen.
SAP = Stable Angina Pectoris
25OHD = 25-hydroxyvitamin D2 and D3

[b] Follow-up measurements of 25OHD were only available from WENBIT patients recruited in Bergen.
