# Supplementary material for: Cosinor modelling of seasonal variation in 25-hydroxyvitamin D concentrations in cardiovascular patients in Norway
Source: Eur J Clin Nutr. 2015 Nov 25;70(4):517–22. doi: 10.1038/ejcn.2015.200 (PMC4827012; doi:10.1038/ejcn.2015.200)
Supplement: Supplementary Table 1 [file ejcn2015200x2.docx]

**ONLINE SUPPLEMENTAL TABLE 1.**Number of subjects according to time from collection of the baseline blood sample to the first and second follow-up blood samples.

|  | **Δ months** | | | | | | | | | | | | | | | |
| --- | --- | --- | --- | --- | --- | --- | --- | --- | --- | --- | --- | --- | --- | --- | --- | --- |
| **Follow-up** | **0** | **1** | **2** | **3** | **4** | **5** | **6** | **7** | **8** | **9** | **10** | **11** | **12** | **13** | **14** | **15** |
| 1^st^ | 43 | 197 | 13 | 2 | 2 | 2 | - | 2 | - | - | - | - | - | - | - | - |
| 2^nd^ | - | - | - | - | - | - | - | 1 | 22 | 34 | 33 | 54 | 50 | 41 | 22 | 10 |

Δ months: The number of months between the time of baseline blood sample collection and the time of the first (n=261) and second (n=267) follow-up.
