# Supplementary material for: Cosinor modelling of seasonal variation in 25-hydroxyvitamin D concentrations in cardiovascular patients in Norway
Source: Eur J Clin Nutr. 2015 Nov 25;70(4):517–22. doi: 10.1038/ejcn.2015.200 (PMC4827012; doi:10.1038/ejcn.2015.200)
Supplement: Supplementary Table 2 [file ejcn2015200x3.docx]

**SUPPLEMENTAL TABLE 2**Classification of subjects according to vitamin D status obtained from measured concentrations of 25-hydroxyvitamin D (25OHD) and annual concentrations derived from different models.

| **Measured 25OHD**  **concentration** | | **Annual 25OHD concentration** | | | **Reclassified** |
| --- | --- | --- | --- | --- | --- |
|  |  | **≥50 nmol/L** | | **<50 nmol/L** |  |
| Linear regression adjusted for season using dummy variables | | | | |  |
| All year (n=4116) | | | n | n | % |
|  | ≥50 nmol/L | | 2566 | 148 | 5.5 |
|  | <50 nmol/L | | 171 | 1231 | 12.2 |
|  | Total | |  |  | 7.8 |
| Dark period (n=2214) | | |  |  |  |
|  | ≥50 nmol/L | | 1309 | 0 | 0.0 |
|  | <50 nmol/L | | 169 | 736 | 18.7 |
|  | Total | |  |  | 7.6 |
| Bright period (n=1902) | | |  |  |  |
|  | ≥50 nmol/L | | 1257 | 148 | 10.5 |
|  | <50 nmol/L | | 2 | 495 | 0.4 |
|  | Total | |  |  | 7.9 |
|  |  | |  |  |  |
| Cosinor model adjusted for age | | | |  |  |
| All year (n=4116) | | | n | n | % |
|  | ≥50 nmol/L | | 2458 | 256 | 9.4 |
|  | <50 nmol/L | | 180 | 1222 | 12.8 |
|  | Total | |  |  | 10.6 |
| Dark period (n=2214) | | |  |  |  |
|  | ≥50 nmol/L | | 1246 | 63 | 5.0 |
|  | <50 nmol/L | | 158 | 747 | 17.5 |
|  | Total | |  |  | 10.0 |
| Bright period (n=1902) | | |  |  |  |
|  | ≥50 nmol/L | | 1212 | 193 | 13.7 |
|  | <50 nmol/L | | 22 | 475 | 4.4 |
|  | Total | |  |  | 11.3 |
|  |  | |  |  |  |
| Cosinor model adjusted for age, gender, smoking and supplement consumption | | | | | |
| All year (n=3590) | | | n | n | % |
|  | ≥50 nmol/L | | 2233 | 158 | 6.6 |
|  | <50 nmol/L | | 231 | 968 | 19.3 |
|  | Total | |  |  | 10.8 |
| Dark period (n=1930) | | |  |  |  |
|  | ≥50 nmol/L | | 1115 | 29 | 2.5 |
|  | <50 nmol/L | | 204 | 582 | 26.0 |
|  | Total | |  |  | 12.1 |
| Bright period (n=1660) | | |  |  |  |
|  | ≥50 nmol/L | | 1118 | 129 | 10.3 |
|  | <50 nmol/L | | 27 | 386 | 6.5 |
|  | Total | |  |  | 9.4 |

Abbreviations and definitions: 25OHD: sum of 25-hydroxyvitamin D2 and D3 (nmol/l).
Dark and bright periods were defined as months of negligible or significant vitamin D synthesis from sun exposure, respectively. Dark period ranges from October through March and bright period from April through September.
